# Contrasting nursery habitats promote variations in the bioenergetic condition of juvenile female red squat lobsters (*Pleuroncodes monodon*) of the Southern Pacific Ocean

**DOI:** 10.7717/peerj.13393

**Published:** 2022-05-04

**Authors:** Fabián Guzmán-Rivas, Marco Quispe, Ángel Urzúa

**Affiliations:** 1 Centro de Investigación en Biodiversidad y Ambientes Sustentables (CIBAS), Universidad Católica de la Santísima Concepción, Concepción, Biobío, Chile; 2 Departamento de Ecología, Facultad de Ciencias, Universidad Católica de la Santísima Concepción, Concepción, Biobío, Chile; 3 Programa de Doctorado en Ciencias con mención en Biodiversidad y Biorecursos, Facultad de Ciencias, Universidad Católica de la Santísima Concepción, Concepción, Biobío, Chile

**Keywords:** Latitudinal variation, Fishery, Body mass, Lipids, Proteins, Glucose, Energy content, Physiological adaptation

## Abstract

The red squat lobster *Pleuroncodes monodon* is an important fishery resource in the Humboldt Current System (HCS). This decapod is exploited in two fishing units: (a) the northern fishing unit (NFU, from 26°S to 30°S) and (b) the southern fishing unit (SFU, from 32°S to 37°S), each of which have an adjacent nursery area that is the source of recruits to replace the exploited adult populations (in the NFU: off the coast of Coquimbo (28°S) and in the SFU: off the coast of Concepción (36°S)). Marked spatial differences in the environmental conditions of the NFU and SFU, and the biogeographic break that exists between these nursery areas (30°S) may promote changes in the bioenergetic condition of new *P. monodon* juveniles. To evaluate this, we analyzed the bioenergetic condition (measured as: body mass, lipids, proteins, glucose, and energy) of new juvenile females in the main nursery areas off the Chilean coast. The juvenile females from the SFU showed a higher body mass than those from the NFU. Consistently, the juvenile females from the SFU had a higher content of lipids, proteins, and glucose than those from the NFU, indicative of higher energy contents and a higher lipid/protein ratio in the south compared to the north. Considering the current overexploitation of this fishery resource in the HCS, it is essential to understand how the bioenergetic condition of juvenile females of *P. monodon* may vary in nursery areas at different latitudes in order to generate sustainable fishery management policies with an ecological approach, designed specifically to each fishing unit. Furthermore, identifying the latitudinal variations of these biochemical compounds in *P. monodon* juveniles can elucidate the geographic origin of red squat lobsters that present a ”better bioenergetic condition” in the HCS, which may significantly benefit sustainable fishing certification processes.

## Introduction

Marine invertebrates with a wide biogeographic range in the Humboldt Current System (HCS, from ∼4°S to ∼45°S) can exhibit spatial variations in key physiological parameters, including their morphometry and biochemical components (Fischer & Thatje, 2016; Montecino & Lange, 2009). These variations can be the result of (i) biotic factors: competition (Cobb & Wahle, 1994), predation (Torossian et al., 2020), parasitism (Powell & Rowley, 2008); (ii) environmental factors: temperature (Fischer & Thatje, 2016), food availability (Pansch et al., 2014), oxygen levels (Paschke et al., 2010); upwelling (Isla et al., 2010), and (iii) anthropogenic factors: fishery pressure (Planque et al., 2010). The HCS is recognized as one of the most productive and fecund marine ecosystems in the world (Montecino & Lange, 2009). Within the HCS, sea water temperature and planktonic food availability and/or productivity (measured as chlorophyll-a) vary with latitude and/or geographic locality. At lower latitudes, warmer waters have proven to lack marked seasonality of biological productivity, while at higher latitudes colder waters have registered a marked seasonality of productivity (Escribano, Fernández & Aranís, 2003; Montecino & Lange, 2009; Thiel et al., 2007). These differences in environmental conditions across a latitudinal gradient have a direct impact on the physiological traits of early life stages of many marine species that support important fishing activities in the HCS (Fischer & Thatje, 2016; Fischer et al., 2009; Guzmán-Rivas et al., 2021a).

As a consequence of spatial differences in key environmental parameters across the latitudinal gradient, marine invertebrate species can exhibit variations in their life history traits (Berryman, 2002; Stearns, 1976), which may have a potential adaptive value in fluctuating environments, such as the HCS. In turn, as an outcome of the main biogeographic break that exists in this area (30°S) (Camus, 2001; Haye et al., 2019), geographically separated populations may also develop local adaptations (Haye et al., 2014; Haye et al., 2019). In addition, marine invertebrate species with the ability to disperse (*i.e.,* free planktonic larvae) have also demonstrated plasticity (Hollander, 2008), and in response to selective pressures from the environment, may show morphological and physiological changes (Haye et al., 2019; Hollander & Butlin, 2010).

Environmental factors, such as temperature and food availability, directly affect metabolism, oxygen consumption, molting cycles, growth, and survival of all phases of the lifecycle of ectothermic marine invertebrates (Le Moullac & Haffner, 2000; Madeira et al., 2018; Woods et al., 2003). Therefore, marine ectotherms tend to present differences in their life history and physiological traits linked to the environmental conditions of their habitat. For example, in the case of some crustaceans from temperate regions, the decapods *Saserma meridies* (Anger, Torres & Nettelmann, 2007), *Romaleon setosus* (Fischer & Thatje, 2016), and *Crangon crangon* (Urzúa & Anger, 2013; Urzúa et al., 2012) presented changes in their fecundity, as well as variations in the size, dry weight, and biochemical composition of their offspring depending on temperature and/or planktonic food availability predominant in the environment.

In general, when fisheries research carry out ecology studies on marine invertebrates, they often overlook morphometric aspects (body mass) and biochemical components (such as lipid, protein, glucose, and energy contents) of juvenile individuals in their recruitment and management models (Smith & Addison, 2003; Thiel et al., 2012; Wehrtmann & Acuña, 2011). In this context, in crustaceans of commercial importance, the “bioenergetic condition” (considered as the body biomass, the biochemical components and lipid/protein ratios) of juveniles may reflect their health status and/or nutritional condition, and subsequently influence the survival and growth rate of adult individuals (*i.e.,* the carry-over effect between ontogenetic phases), which are exploited by commercial fisheries (Smith & Addison, 2003). Also, the lipid/protein ratio is important as in marine animals it indicates the state of health and/or nutritional quality of individuals and allows to discern the metabolic substrate as a source of available energy (lipid form) for fundamental physiological processes (survival, growth, and reproduction) (Aliyu-Paiko, Hashim & Shu-Chien, 2010). Understanding the variations in morphometric and biochemical parameters of juveniles at a large spatial scale throughout the HCS could thus have significant implications for the management and conservation strategies of this fishery resource by helping to identify sources of recruitment and/or nursery grounds (Brosset et al., 2021; Guzmán-Rivas et al., 2021a). Considering that these parameters could also aid in tracing marine resources back to their geographic origins, they could assist in the identification of populations with superior energetic conditions (regarding the traceability concept, see: (Leal et al., 2015; Ricardo et al., 2017; Ricardo et al., 2015)), which should be targeted in the future exploitation of fisheries (FAO, 2019; Olsen & Borit, 2013).

This study focuses on the red squat lobster *Pleuroncodes monodon* H. Milne Edwards, 1837 (Decapoda: Munididae), which has a wide biogeographic distribution throughout the HCS (from Isla Lobos de Afuera (∼7°S) in Perú to Ancud (∼41°S) in Chile) (Haig, 1955; Yannicelli et al., 2012). This squat lobster is a key species in the food chain (Lovrich & Thiel, 2011), as well as an important fishery resource (Zilleruelo, Párraga & Bravo, 2020). *Pleuroncodes monodon* has a complex lifecycle, consisting of a pelagic larval phase characterized by five zoeal stages (Fagetti & Campodonico, 1971) and a benthic juvenile and adult phase (Espinoza et al., 2016; Thiel et al., 2012). It has an extended reproductive cycle, with ovigerous females being present from February to December, and multiple spawning periods (3–4) occurring during its annual cycle (Guzmán, Olavarría & Urzúa, 2016; Thiel et al., 2012). In Chile, *P. monodon* is currently managed using a model that provides fixed annual catches (Wehrtmann & Acuña, 2011) in two fishing units: the northern fishing unit (NFU, from 26°S to 30°S) and the southern fishing unit (SFU, from 32°S to 37°S). Each fishing unit has an adjacent nursery area that is the source of recruits for the exploited adult populations. In the NFU, the nursery area is off the coast of Coquimbo (29°S), and in the SFU it is off the coast of Concepción (36°S) (Gallardo et al., 1994; Roa et al., 1995; Zilleruelo, Párraga & Bravo, 2020). These nursery areas also differ in their environmental factors, coastal geomorphological features, and oceanographic conditions (for characteristics of nursery areas and differences between them: please see “sampling areas” below). There is also a biogeographic break between them at 30°S, which may promote changes in the biochemical components of juvenile *P. monodon* from different nursery areas off the Chilean coast.

In this study, we focused only on juvenile females because they are relevant for structure and population dynamics. These females will produce the offspring that recruit to adult populations (Hamasaki & Kitada, 2008) and maintain the fishing stock (for details on the concept of “’source’ & ’sink”’ in population dynamics of marine crustaceans, see: (Hansen, 2011; Lipcius et al., 1998). In an ecological context, a recent study indicated that the fatty acid profile of *P. monodon* juveniles may vary according to their geographic origin and/or breeding areas throughout the HCS (Guzmán-Rivas et al., 2021a), and that these variations could directly influence the nutritional and/or bioenergetic condition of individuals. This study also highlighted the fact that to help predict the “bioenergetic condition” of subsequent exploitable populations, future research must analyze other key parameters of juvenile individuals, including body mass (*e.g.*, size, weight) and its main biochemical components (*e.g.*, lipids, proteins, glucose, and energy, among others). Within an ecological-fisheries approach, this new information and/or data could also aid in assigning catches to certain populations (*i.e.,* fisheries traceability) and developing sustainable exploitation strategies, especially in areas where this resource has larger sizes and an enhanced bioenergetic condition.

We expected juvenile red squat lobsters to present variations in their body mass and biochemical components across the latitudinal gradient in the HCS, due to differences in key environmental factors (such as temperature and chlorophyll-a) and coastal geomorphological features (*i.e.,* the platform width and depth) of these two nursery areas. Previous studies have identified temporal changes in the biochemical components for each stage of ontogeny from the SFU: eggs (Guzmán et al., 2020), early larval stages (Espinoza et al., 2016; Seguel et al., 2019), and adult individuals (Bascur et al., 2018; Bascur et al., 2017; Guzmán, Olavarría & Urzúa, 2016). However, spatial variations in the biochemical components of juvenile *P. monodon* (onset of benthic phase) from two important breeding areas that are biogeographically separate in the HCS remain unknown. Hence, we analyzed the “bioenergetic condition” (body mass, lipids, proteins, glucose, and energy) of new juvenile females in the main nursery areas off the Chilean coast (NFU: Coquimbo 29°S *vs.* SFU: Concepción 36°S). This study is not only key to evaluate the potential physiological adaptations of juvenile *P. monodon* at different latitudes with contrasting environmental conditions; it is also essential to generate sustainable management policies with an ecological approach. This is also especially relevant to trace the geographic origin of red squat lobsters that may demonstrate a ”better bioenergetic condition” throughout the HCS. In addition, the “bioenergetic condition” of new juvenile females is particularly interesting for the focus of this study because they store the energy necessary for their subsequent first reproduction event and then transfer these energetic reserves to their first broods.

## Materials & Methods

### Sampling areas

Chile is part of the great marine ecosystem referred to as the HCS, from about 18°S (off the coast of Arica) up to about 45°S (off the coast of Chiloe) (Montecino & Lange, 2009). This area is commonly divided into two units: the northern unit and the southern unit. The northern unit is characterized by more stable environmental and oceanographic conditions, lacking marked seasonality due to the presence of interannual El Niño-Southern Oscillation (ENSO) cycles (Thiel et al., 2007). Contrastingly, the southern zone is characterized by marked seasonality in environmental factors (*e.g.*, biological productivity and temperature) with a peak in primary production linked to high sea surface temperatures (SSTs) in the summer (Escribano, Fernández & Aranís, 2003; Figueroa, 2002; Montecino & Lange, 2009). The southern unit is also characterized by having much higher oxygen levels at deep oceanic waters and less intense periods of hypoxia as opposed to the northern unit (Escribano, Fernández & Aranís, 2003; Thiel et al., 2007).

Regarding the coastal geomorphological characteristics of both sampling areas, the continental shelf in the NFU is relatively narrow (∼10 km wide) and the coastal area has little influence from freshwater discharges and lacks big river outlets. On the other hand, the continental shelf in the SFU is wide (∼70 km), deep (∼100 m) and the coasts are characterized by large river mouths, high rainfall, and thus high amounts of incoming fresh water flushing high contents of terrestrial organic matter into the sea (Figueroa, 2002; Figueroa & Moffat, 2000).

**Figure 1:**
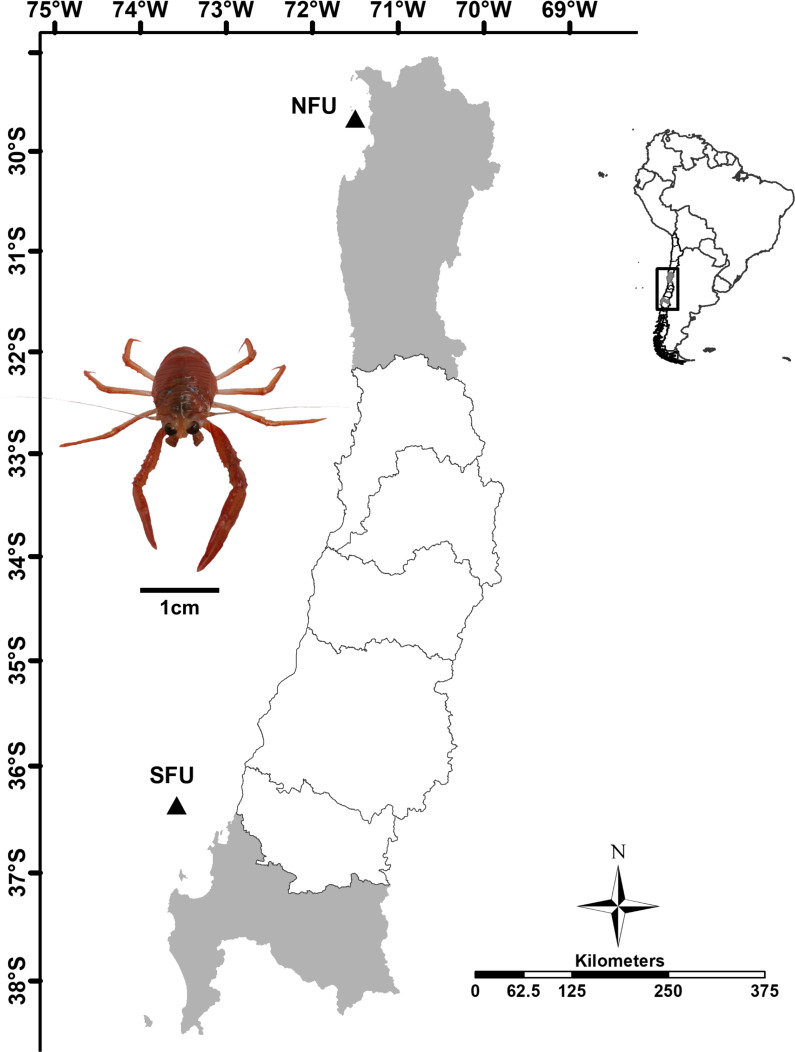
Sampling point of juvenile females of red squat lobster off chilean coast. The figure shows the sampling area in the northern fishing unit (NFU: 29°58′S; 71°38′W) and southern fishing unit (SFU: 36°22′S; 73°35′W) off the coast Coquimbo and Concepción respectively, Chile.

### Collection of juveniles

Juvenile red squat lobsters were sampled as part of the monitoring program for demersal crustacean fisheries from the Pontificia Universidad Católica de Valparaiso and the Instituto de Fomento Pesquero, in collaboration with the fishing company Camanchaca Pesca Sur, S.A. The samples were captured at depths of 80-100 m in May 2016, at the peak abundance of new juveniles (“individuals recently settled in the benthos”, with a similar state of immaturity) in the field, (Roa et al., 1995) off the coasts of Coquimbo (NFU: 29°58′S 71°38′W) and Concepción (SFU: 36°22′S 73°35′W) (Fig. 1) by the Altair I and Trauwun trawling vessels from Camanchaca Pesca Sur, S.A. These nursery areas are very difficult to access, characterized by steep slopes composed of sand and large rocks and are located near long-exploited fishing grounds (Sobarzo & Djurfeldt, 2004; Zilleruelo, Párraga & Bravo, 2020). The samples were kept in containers with dry ice until reaching the Hydrobiological Resources Laboratory of the Universidad Católica de la Santísima Concepción where they were kept frozen at −80°C (Guzmán-Rivas et al., 2021a).

### Environmental factors

The environmental data on sea surface temperatures (SSTs, measured as ^∘^C) and chlorophyll-a concentrations (Chl-a, measured as mg m^−3^) (Table 1) were obtained from the Giovanni website (Earth data, see Acker & Leptoukh (2007)). For the analyses, we used monthly averaged values of each factor (SST, Chl-a) recorded during the year 2016 for the two sampled areas off the coasts of Coquimbo (*i.e.,* NFU) and Concepción (*i.e.,* SFU). Also, the annual average value of each environmental factor was calculated for each study area.

### Preparation of samples for analyses

#### Sex determination and state of immaturity

In the Hydrobiological Resources Laboratory, following Guzmán-Rivas et al. (2021a), the sex of each new juvenile squat lobster was verified using a dissection microscope (SMZ-178, Motic) to assess the morphological differences of the first pair of pleopods (*i.e.,* thick female pleopods *vs.* thin male pleopods) (Gutierrez & Zuñiga, 1977). In turn, the state of immaturity of new juveniles was also determined using a dissection microscope (SMZ-178, Motic) based on two attributes: (i) physiological, following Flores et al. (2020), here a small gonad with a white coloration was described and (ii) functional, defined by Montenegro (2008) as the absence of embryos under the abdomen. From a total of 83 individuals, only new juvenile females were selected, resulting in *n* = 30 new juvenile females from the NFU and *n* = 30 new juvenile females from the SFU. The following methodology and analyses focused only on new juvenile females. New juvenile males were not considered in this study because insufficient male samples were obtained from the field for analyses (*n* = 9; total = ∼90% female *vs.* ∼10% male).

**Table 1:** Environmental data average (sea surface temperature, SST; chlorophyll-a, Chl-a) of sampling area in the northern fishing unit (NFU; 29°58′S; 71°38′O) and southern fishing unit (SFU; 36°22′S; 73°35′O) off the coast Coquimbo and Concepción respectively, Chile.

Environmental variable	Fishing unit	Jan	Feb	Mar	Apr	May*	Jun	Jul	Aug	Sept	Oct	Nov	Dec
SST (°C)	NFU	18.67 ± 0.2	18.24 ± 1.66	18.38 ± 0.82	16.41 ± 1.46	15.62 ± 0.25	13.79 ± 0.61	14.38 ± 0.51	13.52 ± 0.06	13.72 ± 0.24	14.19 ± 0.57	16.41 ± 1.55	17.2 ± 1.06
	SFU	16.79 ± 1.29	15 ± 0.53	14.17 ± 0.11	14.18 ± 0.23	14.84 ± 0.08	13.72 ± 0.74	12.61 ± 0.28	12.79 ± 0.42	13.09 ± 0.25	13.44 ± 0.35	13.23 ± 1.2	14.76 ± 0.67
Chl-a (mg m^3^)	NFU	1.15 ± 0.61	0.78 ± 0.31	0.97 ± 0.43	0.54 ± 0.17	0.73 ± 0.03	1.16 ± 0.44	0.56 ± 0.16	1.86 ± 0.96	1.13 ± 0.36	1.75 ± 1.25	1.14 ± 0.82	1.19 ± 0.29
	SFU	1.81 ± 0.79	1.9 ± 0.77	2.62 ± 0.71	0.82 ± 0.19	0.78 ± 0.04	1.38 ± 0.93	0.87 ± 0.15	1.57 ± 0.56	2.19 ± 1.24	0.63 ± 0.12	4.23 ± 3.16	1.87 ± 0.52

**Notes.**

The event sampling month is indicated with an asterisk (May).

#### Size and body mass

A caliper (±0.001 mm) was used to measure cephalothorax length (CL) of new juvenile females. The CL was measured from the posterior margin of the cephalothorax to the base of the rostral spine. Then, as described in detail by Guzmán-Rivas et al. (2021a), all internal organs (*i.e.,* viscera) and muscle were removed from each new juvenile female. Due to the small size of the specimens, we mixed all of the internal organs and referred to this as “viscera” (which was mainly composed of hepatopancreas (∼90%), stomach and ovary (∼10%)) (Guzmán-Rivas et al., 2021b; Mantel, 1983; Subramoniam, 2017). The storage and subsequent weighing of samples was carried out following the same protocol as described by Guzmán-Rivas et al. (2021b). The viscera and muscle samples (20 mg of dry weight (DW)) were then used to analyze the biochemical components (lipid, protein, glucose, and energy contents).

### Biochemical components

For the biochemical analysis, the methodologies that were recently described in detail by Guzmán et al. (2020), Guzmán-Rivas et al. (2021a) and Lazo-Andrade et al. (2021) were used.

#### Lipid content

For the lipid extraction, the dry weight (*i.e.,* 20 mg) of each sample (viscera and muscle) was mixed with dichloromethane: methanol (2:1) solvent, following the method described by Folch, Lees & Sloane Stanley (1957), Cequier-Sánchez et al. (2008), and Saini et al. (2021). Then, an ultrasonic bath (MRC, AC-120H) was used to incubate the samples. After that, each sample was mixed with potassium chloride (0.88%) and homogenized in a vortex (Select Vortexer, model SBS100-2). Each sample was centrifuged (Boeco, model S-8) and the lower and/or organic phase was collected and dried in a sample concentrator (109A YH-1, Glas-Col) to register their DW. Finally, the total amount of lipids in the samples was calculated as the difference between the DW of the empty amber vials and their DW after the lipid collection.

#### Protein content

The protein content was measured using the colorimetric method (Bradford, 1976) and modified for microplates. For this, dry tissue samples were mixed and homogenized with ultra-pure water and sonicated for 10 min. Then, an aliquot of the homogenate was mixed with the Bradford reagent. Subsequently, this mixture was transferred into 96-well microplates. Highly concentrated samples were diluted with a dilution solution reagent. After 15 min. at room temperature, the absorbance of each sample was measured at a wavelength of 595 nm. A calibration curve was built using the absorbance of different protein concentrations (from 0.1 mg mL^−1^ to 1.5 mg mL^−1^) of bovine serum albumin (BSA) diluted with the dilution solution. These measurements were used to determine the DW of proteins within 20 mg.

#### Glucose content

The glucose content was determined with a kit (Spinreact) that implements the colorimetric method (Tietz, 1995) modified for microplates. In brief, a dry sample was rehydrated and mixed with ultra-pure water. Then, a small aliquot was mixed with a working reagent. In addition, a glucose standard was prepared with a working reagent and an organic compound glucose. The samples were incubated for 20 min. at room temperature. The absorbance was then measured at a wavelength of 490 nm. The absorbance was measured relative to a blank sample that contained only the working reagent. The net glucose content for each 20 mg tissue sample was then obtained by dividing the absorbance of the sample by the absorbance of the standard.

#### Energy content

The energy content of each sample was measured in Joules (J) and estimated using the energy bioequivalents of the analyzed biochemical components, with the following conversion coefficients: 1 mg lipids = 39.54 J, 1 mg proteins = 23.69 J, and 1 mg glucose = 17.15 J (Guzmán et al., 2020; Lazo-Andrade et al., 2021; Urzúa et al. 2012; Winberg, 1971). Total energy was considered as the sum of the energy contributed by each of these biochemical components.

#### Lipid/Protein ratio

From the previous estimations, the lipid/protein (L/P) ratios were calculated by dividing the lipid content (mg g^−1^ DW) by the protein content (mg g^−1^ DW) of each sample. The L/P ratio was used as a proxy for the nutritional condition of the juvenile females in relation to the environmental conditions present in the nursery areas (NFU and SFU). Additionally, to make a global comparison of the L/P ratio (used as an index of the nutritional condition) in species of juvenile decapods from different geographical regions, values of this index were obtained from the Web of Science database of bibliographic references.

### Statistical analysis

Statistical analyses were performed using parametric, non-parametric, and multivariate methods (Sokal & Rohlf, 1995; Zuur, Leno & Smith, 2007), which were carried out in the SigmaStat 4.0 (StatSoft), Brodgar 2.7.5 with R-mgcv package (Highland Statistics Ltd.) and PRIMER 6 & PERMANOVA + software. For environmental factor analyses, a generalized additive model (GAM) with a Gaussian distribution was used to explain the nature of the smoothing function (S) of each environmental factor (SST, Chl-a) in the two nursey areas and/or localities (NFU and SFU) throughout the year. Also, a parametric test (paired *t*-test) was run to measure the differences for each environmental parameter between nursery areas over the annual period of study. In turn, to evaluate differences in the bioenergetic condition (DW, lipid, protein, glucose, and energy contents) of new juvenile females between nursery areas a *t*-test was carried out. Prior to the *t*-test analyses of the bioenergetic condition, assumptions of homogeneity (Levene’s test) and normality (Shapiro–Wilk test) tests were performed using a significance level of 0.05. We considered natural logarithm transformation for DW data and square root transformation for L/P ratio data (viscera and muscle) to meet with the previously described assumptions; and when the assumptions were not met, the non-parametric test (Mann–Whitney rank sum test (U)) was used. Altogether, variations in the “bioenergetic condition” of the viscera and muscle of new juvenile females related to the ”locality factor” (NFU *vs.* SFU) were assessed with a permutational multivariate analysis of variance (PERMANOVA) with a Euclidean distance matrix and square root transformation. Lastly, a principal coordinate analysis (PCoA) was performed to graphically display the differences found in the PERMANOVA.

## Results

### Environmental parameters

**Figure 2:**
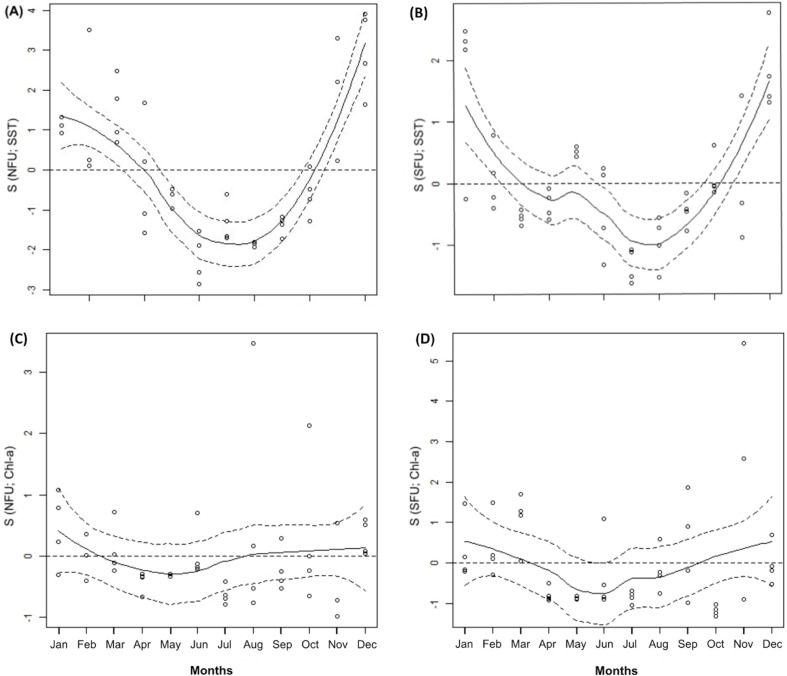
Generalized additive model of environmental parameters (sea surface temperature and chlorophyll-a). The figure shows monthly variation of sea surface temperature (SST) (A and D) and chlorophyll-a (Chl-a) (B and C) from northern fishing unit (NFU; 29°58′S; 71°38′O) and southern fishing unit (SFU; 36°22′S; 73°35′O) off the coast Coquimbo and Concepción respectively, Chile. Continuous line: estimated smoothing function; Segmented line: 95% confidence intervals; dots represent mean values for each month. Months = 12; January to December.

Throughout the annual period of study, the SFU showed lower average SSTs than the NFU (14.05 ± 0.77 °C *vs.* 15.97 ± 1.07 °C; SFU and NFU, respectively) (t_1,11_ = 4.928; *P* < 0.001; Table 1). Both localities were characterized by a notorious peak in the austral warm season (*i.e.,* December and January) (Figs. 2A and 2B; GAM, S function). On the other hand, the Chl-a levels in both localities remained relatively stable throughout the year (SFU: 1.69 ± 1.26 mg m^3^ *vs.* NFU: 1.25 ± 0.82 mg m^3^) (t _1,11_ = −1.879; *P* = 0.087; Table 1), with only slight variations in their concentrations (Figs. 2C and 2D; GAM, S function).

### Size and Body mass

**Figure 3:**
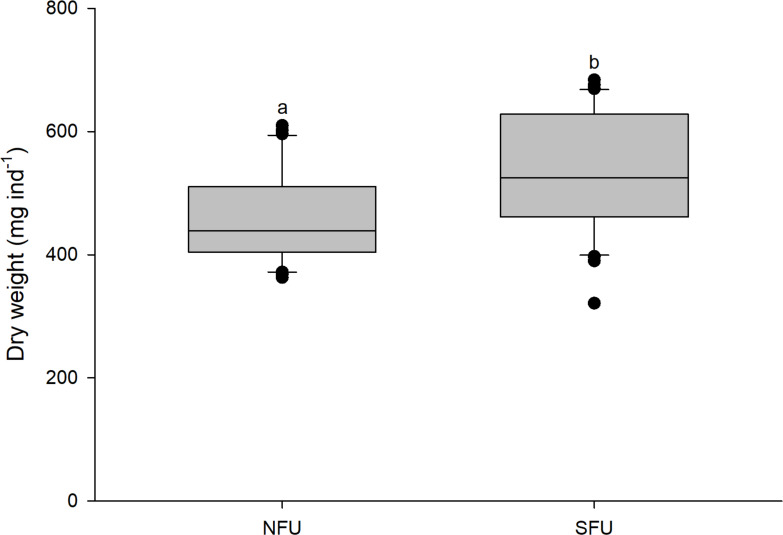
Differences in body mass of juvenile females of the red squat lobster. The figure shows differences in dry weight (mg ind^−1^) of juvenile females of the red squat lobster (*Pleuroncodes monodon*) from the northern fishing unit (NFU) and the southern fishing unit (SFU) off the coast Coquimbo and Concepción respectively, Chile. The figure shows average values and standard deviations. Different lowercase letters indicate significant differences.

The size (measured as CL) of new juvenile females was similar in the two sampled nursery areas (SFU: 19.2 ± 1.45 mm *vs.* NFU: 19.9 ± 1.45 mm) (t_1,58_ = 1.880; *P* = 0.065). However, the new juvenile females from the SFU showed a higher body mass (quantified as DW) than those from the NFU (SFU: 532.4 ± 97.2 mg *vs.* NFU: 461.7 ± 75.94 mg; Fig. 3) (t_1,58_ = −3.126; *P* < 0.01).

### Lipid content

**Figure 4:**
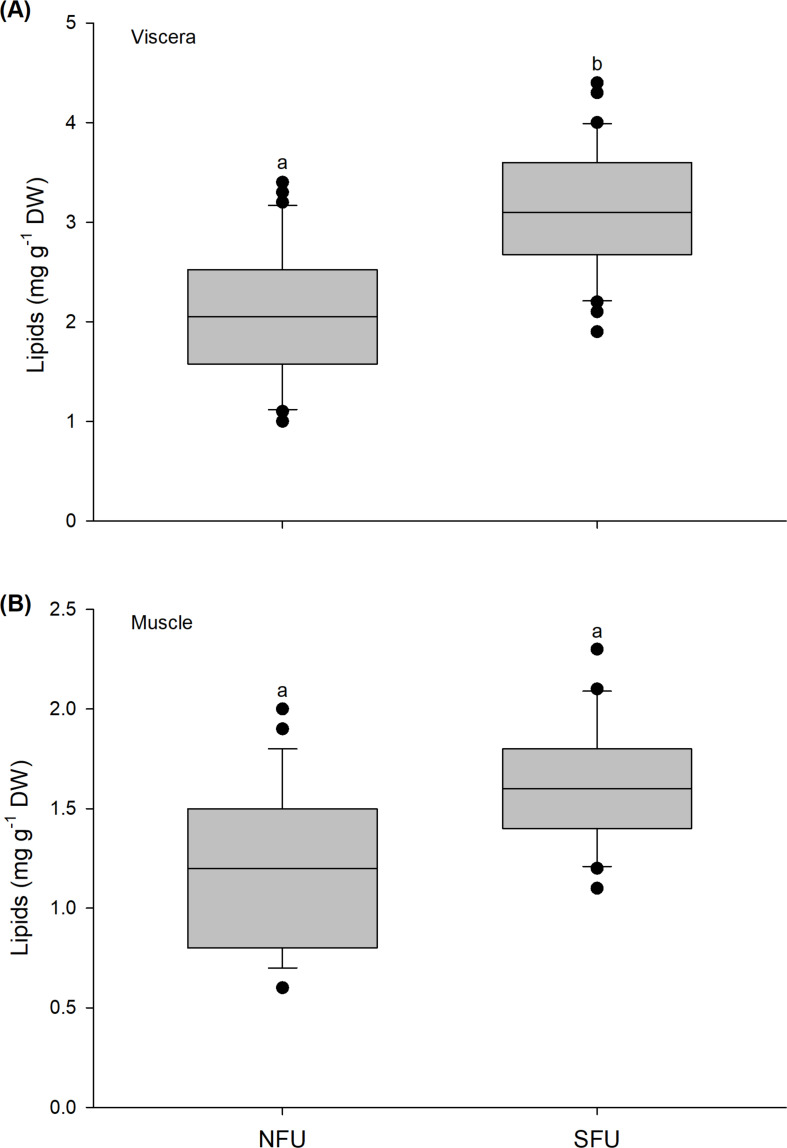
Differences in the lipid content of juvenile females of the red squat lobster. The figure shows biochemical differences in the lipid content of the viscera (mg g^−1^ DW) (A) and muscle (mg g^−1^ DW) (B) in juvenile females of the red squat lobster (*Pleuroncodes monodon*) from northern fishing unit (NFU) and southern fishing unit (SFU) off the coast Coquimbo and Concepción respectively, Chile. The figure shows average values and standard deviations. Different lowercase letters indicate significant differences.

The lipid content in the viscera of new juvenile females varied significantly between the two nursery areas (t_1,58_ = −6.037; *P* < 0.001). The viscera of the new juvenile females from the SFU (3.11 ± 0.65 mg g^−1^ DW) had a higher lipid content than those from the NFU (2.1 ± 0.65 mg g^−1^ DW) (Fig. 4A). In contrast, the lipid content in the muscle of new juvenile females did not statistically vary between the nursery areas (Mann–Whitney U_1,58_ = 435.5; *P* = 0.835), with similar average values between the two localities (SFU: 1.64 ± 0.29 mg g^−1^ DW *vs.* NFU: 1.29 ± 0.42 mg g^−1^ DW) (Fig. 4B).

### Protein content

**Figure 5:**
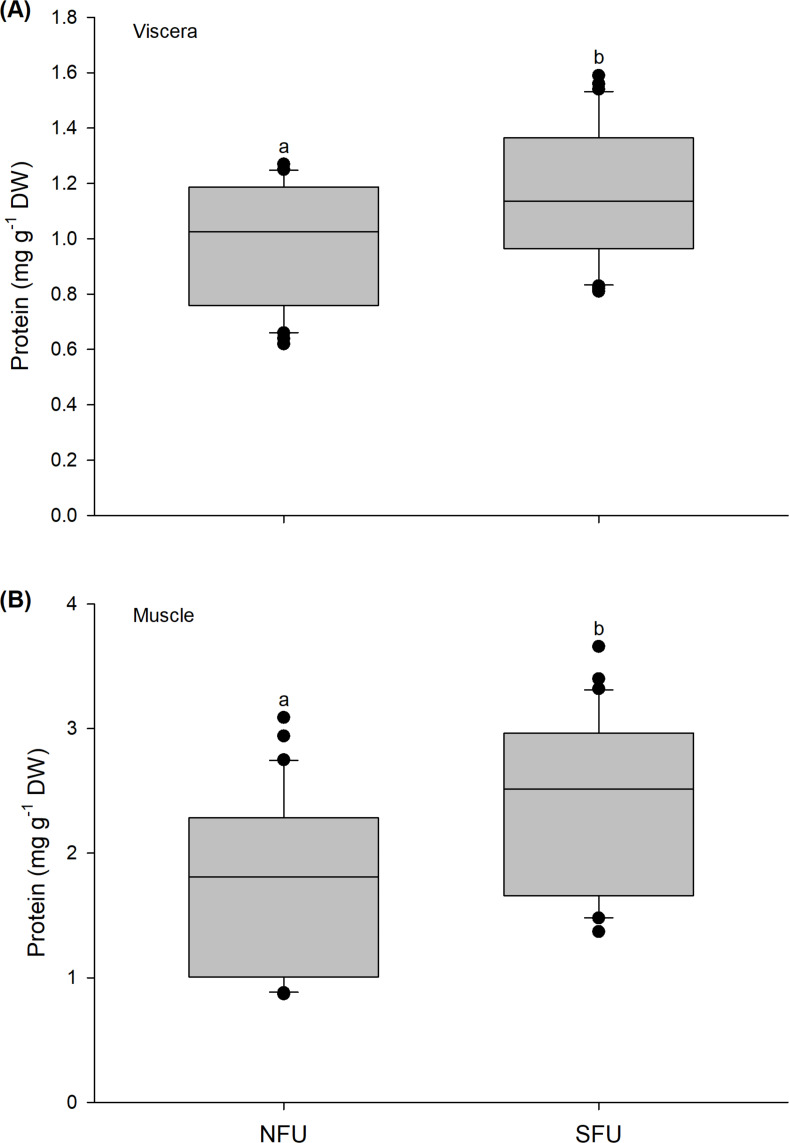
Differences in the protein content of the juvenile females of red squat lobster. The figure shows biochemical differences in the protein content of the viscera (mg g^−1^ DW) (A) and muscle (mg g^−1^ DW) (B) in juvenile females of the red squat lobster (*Pleuroncodes monodon*) from northern fishing unit (NFU) and southern fishing unit (SFU) off the coast Coquimbo and Concepción respectively, Chile. The figure shows average values and standard deviations. Different lowercase letters indicate significant differences.

The protein content in the viscera of new juvenile females also varied significantly between the nursery areas (Mann–Whitney U_1,58_ = 292; *P* = 0.02). A high protein content was detected in new juvenile females from the SFU (1.15 ± 0.24 mg g^−1^ DW) compared to those from the NFU (0.99 ± 0.21 mg g^−1^ DW) (Fig. 5A). A similar tendency was also observed for protein contents quantified in the muscle, with significant differences between nursery areas (Mann–Whitney U_1,58_ = 258.5; *P* < 0.01), and again, a higher protein content in the new juvenile females from the SFU (2.4 ± 0.71 mg g^−1^ DW) than in those from the NFU (1.79 ± 0.69 mg g^−1^ DW) (Fig. 5B).

### Glucose content

**Figure 6:**
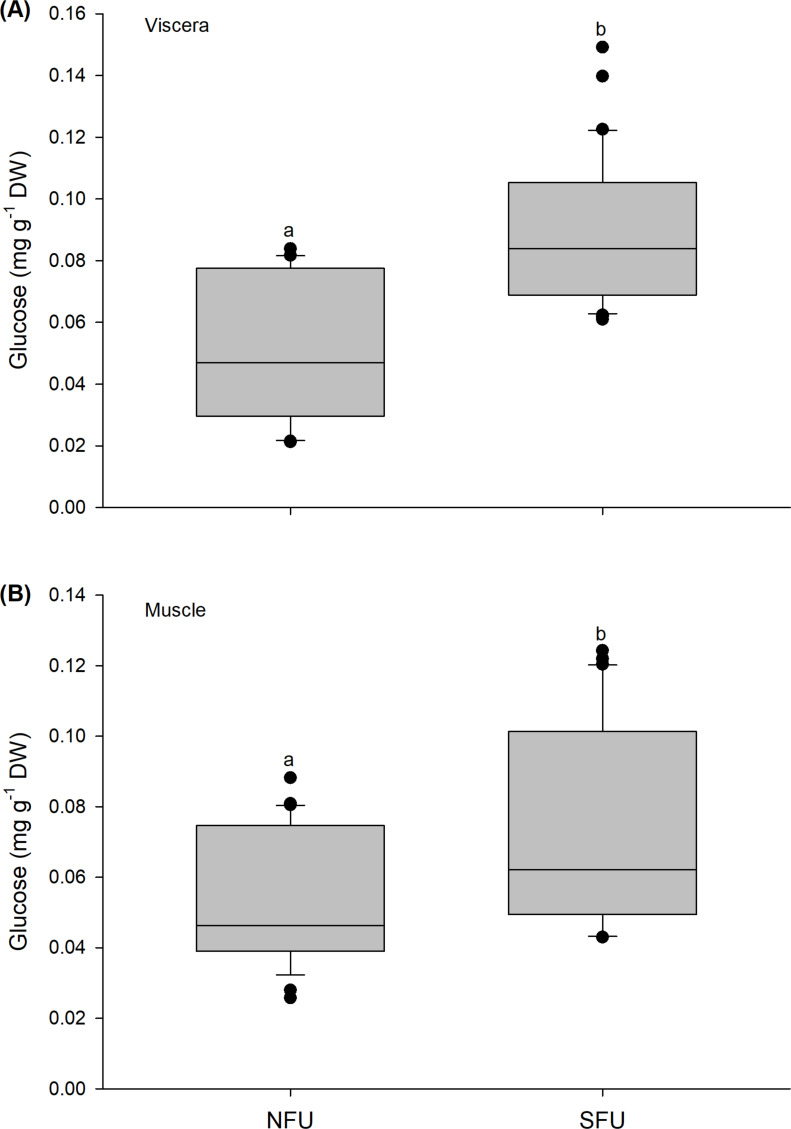
Differences in the glucose content of the juvenile females of red squat lobster. The figure shows biochemical differences in the glucose content of the viscera (mg g^−1^ DW) (A) and muscle (mg g^−1^ DW) (B) in juvenile females of the red squat lobster (*Pleuroncodes monodon*) from northern fishing unit (NFU) and southern fishing unit (SFU) off the coast Coquimbo and Concepción respectively, Chile. The figure shows average values and standard deviations. Different lowercase letters indicate significant differences.

The glucose content quantified in the viscera of new juvenile females showed a similar tendency as observed above, with significant variations between locations (Mann–Whitney U_1,58_ = 136.5; *P* < 0.01). Higher average values were found in new juvenile females from the SFU (0.09 ± 0.02 mg g^−1^ DW) than in those from the NFU (0.05 ± 0.02 mg g^−1^ DW) (Fig. 6A). Significant differences were also recorded for the muscle in the two locations (Mann–Whitney U_1,58_ = 255; *P* < 0.01), and again, the new juvenile females from the SFU (0.07 ± 0.03 mg g^−1^ DW) had a higher amount of glucose compared to those from the NFU (0.05 ± 0.02 mg g^−1^ DW) (Fig. 6B).

**Figure 7:**
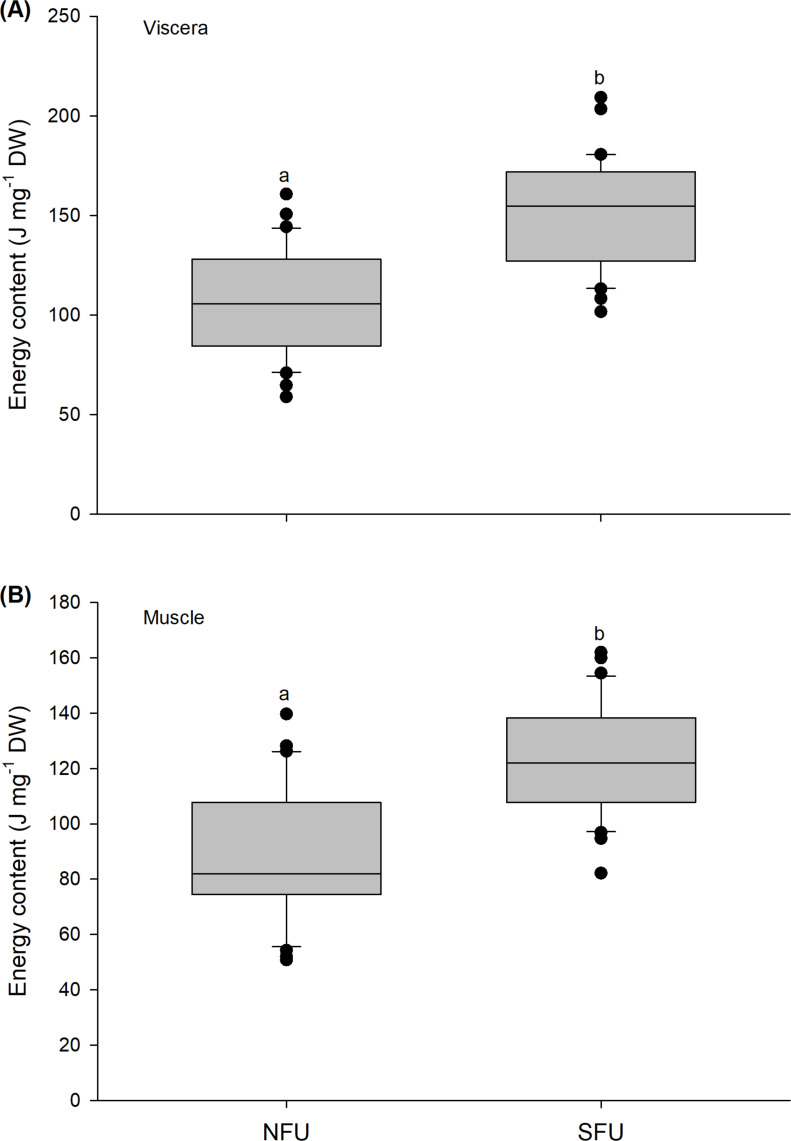
Differences in the energy content of the juvenile females of red squat lobster. The figure shows differences in the energy content of the viscera (J mg^−1^ DW) (A) and muscle (J mg^−1^ DW) (B) in juvenile females of the red squat lobster (*Pleuroncodes monodon*) from northern fishing unit (NFU) and southern fishing unit (SFU) off the coast Coquimbo and Concepción respectively, Chile. The figure shows average values and standard deviations. Different lowercase letters indicate significant differences.

### Energy content

The energy content estimated from the evaluated viscera and muscle of new juvenile females showed significant differences between the two nursery areas (viscera: t_1,58_ = −6.476; *P* < 0.001; muscle: t_1,58_ = −5.712; *P* < 0.001). For the viscera, new juvenile females from the SFU (151.9 ± 27.4 J mg^−1^ DW) showed a higher energy content compared to those from the NFU (107.1 ± 26.12 J mg^−1^ DW) (Fig. 7A). Similarly, the muscle of new juvenile females from the SFU (122.8 ± 19.57 J mg^−1^ DW) presented a higher energy content than those from the NFU (90.43 ± 24.11 J mg^−1^ DW) (Fig. 7B).

### Lipid/protein ratio

**Figure 8:**
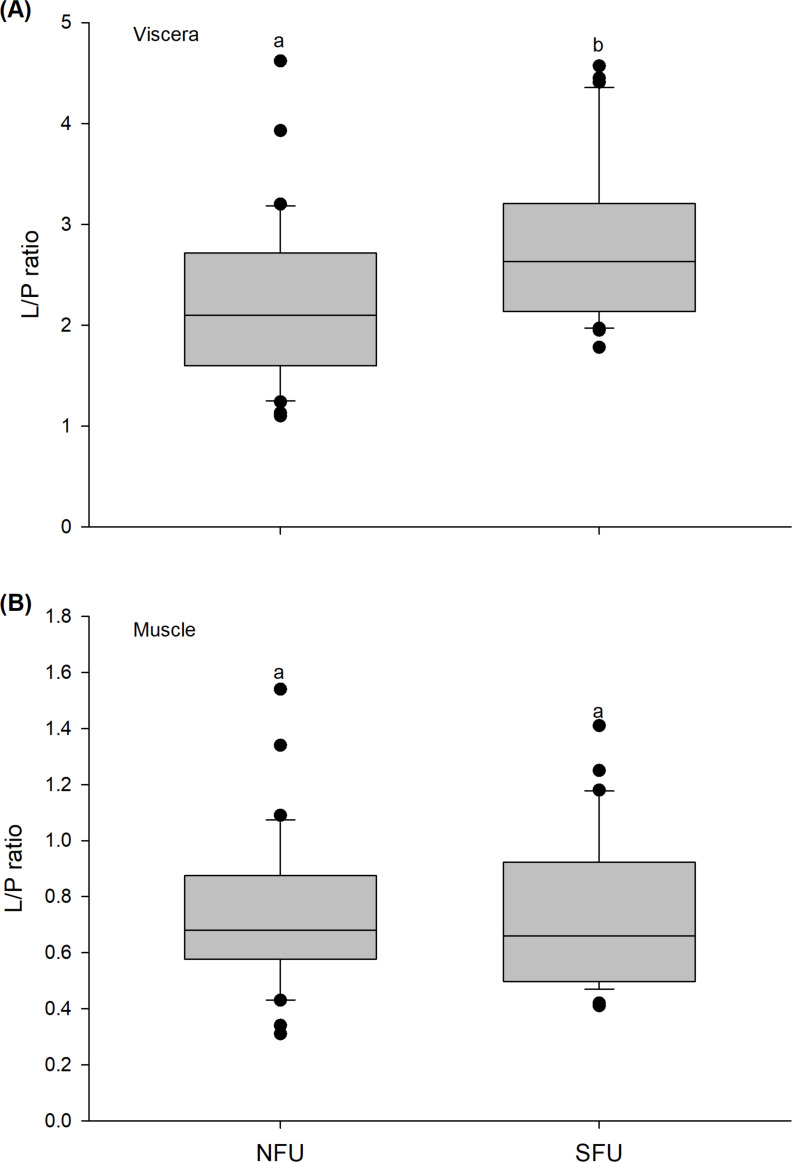
Differences in the Lipid/Protein (L/P) ratio of the juvenile females of red squat lobster. The figure shows L/P ratios of the viscera (A) and muscle (B) of juvenile females of the red squat lobster (*Pleuroncodes monodon*) from northern fishing unit (NFU) and southern fishing unit (SFU) off the coast Coquimbo and Concepción respectively, Chile. The figure shows average values and standard deviations. Different lowercase letters indicate significant differences.

The L/P ratio of the viscera of new juvenile females varied significantly between the two nursery areas (t_1,58_ = −2.971; *P* < 0.005), with higher L/P values detected in juvenile females from the SFU (2.8 ± 0.78) compared to those from the NFU (2.23 ± 0.82) (Fig. 8A). On the other hand, the L/P ratio of the muscle of juvenile females did not vary significantly between the localities (t_1,58_ = −0.372; *P* = 0.711), and showed quite similar average values (SFU: 0.75 ± 0.28 *vs.* NFU: 0.72 ± 0.27) (Fig. 8B).

### Multivariate analysis of bioenergetic condition

**Figure 9:**
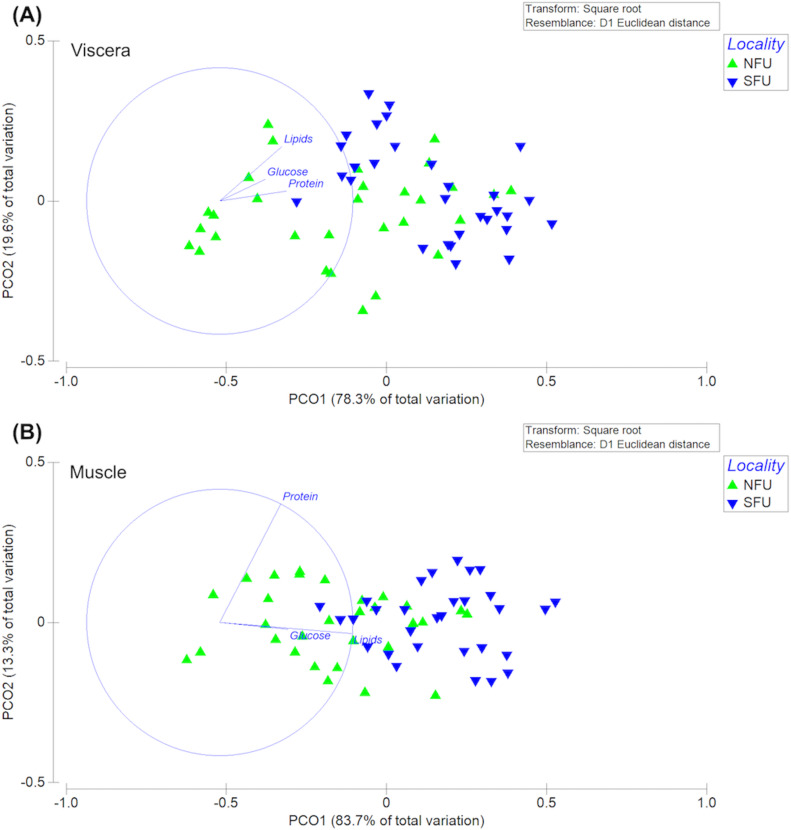
Principal coordinates analysis (PCoA) of biochemical profile of the juvenile females of red squat lobster. The figure shows the biochemical profile (*i. e.,* lipid, protein and glucose) of the viscera (A) and muscle (B) of the red squat lobster (*Pleuroncodes monodon*) from the northern fishing unit (NFU) and the southern fishing unit (SFU) off the coast Coquimbo and Concepción respectively, Chile.

The permutational multivariate analysis of variance (PERMANOVA) of the bioenergetic condition of the viscera of new juvenile females showed statistically significant differences between the two nursery areas (*F*_1,58_ = 30.47; P-pseudo = 0.001). The PC1 axis of the principal coordinate analysis (PCoA) explained 83.7% of the variation, while the PC2 axis explained only 13.3% of the data variation (Fig. 9A). In this case, the differences measured in the bioenergetic condition of new juvenile females could be explained by the lipid content in the viscera. On the other hand, the PERMANOVA of the muscles’ bioenergetic condition also showed significant differences between the two localities (*F*_1,58_ = 15.01; P-pseudo = 0.001). The PC1 axis of the principal coordinate analysis (PCoA) explained 78.3% of the variation, while the PC2 axis explained only 19.6% of the data variation (Fig. 9B). Here, the differences in the bioenergetic condition of new juvenile females could be explained by the protein content in the muscle.

## Discussion

In our study, we found variations in the body mass (dry weight) and biochemical components (lipids, proteins, glucose, and energy) of new juvenile *P. monodon* females in two nursery areas (NFU and SFU). We suggest that these differences may reflect potential adaptive physiological responses of *P. monodon* juveniles to local environmental factors (mainly water temperature) (Anger, Torres & Nettelmann, 2007) and biogeographic features (*i.e.,* coastal geomorphological characteristics) (Ansari, Farshchi & Faniband, 2010; Calvo et al., 2018; Camus, 2001; Haye et al., 2019), which differ between these two nursery areas and/or locations (29°S *vs.* 36°S). The physiological capacity of juvenile red squat lobsters to adapt to the local environment may result in a more flexible, dynamic, and successful recruiting of juvenile individuals to adult populations (Floreto, Bayer & Brown, 2000). In particular, identifying variations in bioenergetic condition (as an integrated concept: measured as the body mass and biochemical components) of juvenile female red squat lobsters at different latitudes and/or localities of the HCS may help to predict and estimate the abundance of adult populations (*i.e.,* exploitable biomass), which are exploited by commercial fisheries in the HCS. Moreover, for sustainable fishing certification processes of crustaceans (Naylor et al., 2021; Nyiawung, Raj & Foley, 2021), the detection and description of site-specific attributes, such as biochemical components (used as biological tracers: (Fonseca et al., 2022; Shilla & Routh, 2017) of red squat lobster stocks will aid in tracing this fishery resource back to its geographic origin or source population. Furthermore, identifying which stock is of higher quality in terms of its bioenergetic condition could greatly benefit the sustainable management of these fisheries in the HCS (Smith & Addison, 2003).

Many commercial decapod species of marine temperate regions (*e.g.*, *Crangon crangon, Romaleon setosum*, (Fischer et al., 2009; Urzúa & Anger, 2013), including the red squat lobster *P. monodon*, have a wide geographical distribution range across the climatic gradient, which is frequently characterized by fluctuating and contrasting environmental conditions, both spatially (between latitudes) and temporally (between seasons) (Montecino & Lange, 2009; Thiel et al., 2007). Hence, they have evolved to respond physiologically to environmental changes and selective pressures, which can be considered as biochemical adaptive responses to different environmental conditions (Calvo et al., 2013; Floreto, Bayer & Brown, 2000; Hochachka & Somero, 2002). For instance, in marine ectotherms at higher or warmer temperatures their development time is shorter, with faster growth rates and metabolism, while body weights are lower and contain less lipids and proteins, when compared to specimens that develop at lower or colder temperatures (Fischer & Thatje, 2016; Tropea, Stumpf & López, 2015; Wang & Stickle, 1988). Body mass, in particular, is one of the most important traits of an organism in terms of physiology and fitness traits (Rosa & Nunes, 2003a) and a positive correlation between latitude and body mass has been well documented (Anger & Hirche, 1990; Rosa & Nunes, 2003b; Rotllant et al., 2014). In our study, juvenile females from the SFU (a higher latitude) showed a higher total body mass than those from the NFU (at lower latitudes). Hence, the general trend to increase body size as latitude increases (Rotllant et al., 2014) holds true for the juvenile females of *P*. *monodon* studied herein. This tendency has also been reported for other decapod crustacean species from high latitudes and/or temperate regions (*e.g.*, *Uca uruguayensis*, (Rosa & Nunes, 2003b); *Plesionika edwardsii*, (Gonzalez et al., 2016).

**Table 2:** Lipid/protein (L/P) ratio of decapod crustacean species living in different regions of the world.

**Specie**	**L/P ratio**	**Tissue analyzed**	**Distribution range**	**Reference**
*Pleuroncodes monodon*	1.2–1.56	Viscera + muscle	Humboldt Current System (7°S—37°S)	Present study
*Maja brachydactyla*	∼0.45	Whole body	Atlantic coast (55°N –44°N)	Andrés et al. (2008)
*Sesarma meridies*	∼0.31	Whole body	Freshwater, Jamaica (18°N –17°N)	Anger, Torres & Nettelmann (2007)
*Farfantepenaeus paulensis*	∼0.15	Whole body	Southwest Atlantic waters, shelf waters, estuarine (14°S–18°S)	Lemos, Phan & Alvarez (2001)
*Metapenaeus affinis*	∼0.23^*^	Whole body	Indo-Pacific: from Persian Gulf and Arabian Sea to South China Sea and Hawaii (31°N–18°S)	Ansari, Farshchi & Faniband (2010)
*Cherax quadricarinatus*	∼0.21	Whole body	Freshwater, Australia, Papua New Guinea (7°S–27°S)	Calvo et al. (2018)
*Homarus americanus*	0.08–0.12^*^	Whole body	North Atlantic coast (60°N–33°N)	Floreto, Bayer & Brown (2000)
*Jasus edwardsii*	0.06–0.13^*^	Whole body	Indo-West Pacific: Australia, New Zealand and Chatham Island (31°S –51°S)	Ward & Carter (2009)
*Maja brachydactyla*	0.03–0.07^*^	Body without carapace	East coast of the Atlantic ocean (55°N–44°N)	Domingues et al. (2012)
*Cherax quadricarinatus*	0.41–0.85^*^ 0.13 –0.42^*^	Hepatopancreas Pleon	Freshwater, Australia, Papua New Guinea (7°S–27°S)	Calvo et al. (2013)
*Neocaridina heteropoda heteropoda*	0.12–0.19^*^	Whole body	Freshwater, China, Taiwan, Vietnam (46°N–10°N)	Tropea, Stumpf & López (2015)
*Callinectes sapidus*	0.19–0.2^*^	Whole body	Northwest Atlantic Ocean (47°N–36°S)	Wang & Stickle (1988)
*Nephrops norvegicus*	∼3.4^*^ ∼0.13^*^ ∼0.52^*^	Hepatopancreas Muscle Ovary	Eastern Atlantic coast, Mediterranean Sea coast (69°N–30°N)	Rosa & Nunes, (2003a) Rosa & Nunes, (2003b)
*Parapenaeus longirostris*	∼4.45^*^ ∼0.14^*^ ∼0.49^*^	Hepatopancreas Muscle Ovary	Atlantic Ocean (66°N–17°S)	Rosa & Nunes, (2003a) Rosa & Nunes, (2003b)
*Aristeus antennatus*	∼4.68^*^ ∼0.13^*^ ∼0.54^*^	Hepatopancreas Muscle Ovary	Eastern Atlantic, Mediterranean sea (43°N–46°S)	Rosa & Nunes, (2003a) Rosa & Nunes, (2003b)
*Maja squinado*	0.62^*^	Whole body	Northwest Atlantic, Mediterranean sea (58°N–19°S)	Rotllant et al. (2014)
*Hyas araneus*	∼5	Whole body	Intertidal, Atlantic Ocean, North Sea (78°N–38°N)	Anger & Hirche (1990)

**Notes.**

* Values calculated from the lipid and protein data presented in each article (for details see reference).

The viscera (or all of the hepatopancreas) are also essential among crustaceans due to the energy storage capacity of these organs in the form of lipids (Chang & O’connor, 1983; Han et al., 2015). Also, the viscera provide the nutrients necessary for reproduction (*e.g.*, gonad development and egg production) and can metabolize and assimilate a diversity of essential nutrients for the entire organism (Daly, Eckert & Long, 2020; Garcia-Guerrero, Villarreal & Racotta, 2003). In contrast, the analyzed muscle tissues of new juvenile females in both locations presented higher protein contents than the viscera because the muscle is the main protein storage organ (Haye et al., 2010; Haye et al., 2014). In juvenile *P. monodon* females, a higher lipid content in the viscera, available as bioenergetic fuel, is fundamental to support the high energy cost of their first reproductive event (characterized by egg laying and incubation), which occurs during the austral winter, with cold water-temperatures and low food availability in the environment (Guzmán-Rivas et al., 2021b; Guzmán, Olavarría & Urzúa, 2016; Seguel et al., 2019; Thiel et al., 2012).

Juvenile females of *P. monodon* from the SFU presented a higher energy content, and also a consistently higher L/P ratio than those from the NFU. This greater accumulation of energy reserves in the form of lipids in the juvenile females from the SFU is mainly related to the higher lipid content present in their tissue, most likely due to a physiological capacity to store greater amounts of lipids at low temperatures (Green et al., 2014; Urzúa & Anger, 2013). A similar physiological response has been described for other juvenile decapod crustaceans from high and subpolar latitudes (for details see Table 2), climatic regions characterized by a marked seasonality of planktonic food and cold-water temperatures. In an ecophysiological context, these predominant environmental conditions may promote a higher accumulation of energy reserves in marine invertebrates, helping them to face low temperatures and periods of food shortage during a prolonged winter, which frequently occurs at these latitudes (Fischer et al., 2009; Weiss et al., 2012). Also, the upwelling of nutrient rich cold waters along the southern latitude may increase productivity (Montecino & Lange, 2009; Thiel et al., 2007), which fuels the marine food web and, in combination with lower temperatures, leads to a greater accumulation of nutrients and an improved bioenergetic condition of marine organisms along the southern coast of Chile (Woods et al., 2003). In this way, knowing these biochemical tracers (*i.e.,* lipids, protein, glucose and ratios) along a latitudinal gradient can allow to trace the place of origin of individuals (Leal et al., 2015; Olsen & Borit, 2013; Ricardo et al., 2015), and identify recruitment and/or nursery areas as a source of new individuals for the adult population (Hansen, 2011; Lipcius et al., 1998), as well as identifying which fishing unit has the best biochemical conditions (for example, a high lipid/protein ratio).

In the context of species interactions in the food web realm, hake (*Merluccius gayi*), the South American sea lion (*Otaria flavescens*), and squid (*Dosidicus gigas*) are described as the main predators of *P*. *monodon* (Cubillos, Rebolledo & Hernandez, 2003; Sarmiento-Devia et al., 2020). Although these predators show a certain degree of mobility, their trophic habits and lifestyles are closely linked to specific feeding areas and/or locations (Payá & Ehrhardt, 2005; Sarmiento-Devia et al., 2020; Sepúlveda, Carrasco & Quiñones, 2021), where they consume prey with a high energy content, such as the red squat lobster (Sarmiento-Devia et al., 2020; Wehrtmann & Acuña, 2011). Hence, the spatial variations in the bioenergetic condition of *P. monodon* may modify the energy transfer to higher trophic levels (Lovrich & Thiel, 2011) and impact the nutritional status of these predators, which, in turn, are also important fishery resources (*M*. *gayi* and *D*. *gigas*) in the HCS (Alarcón-Muñoz, Cubillos & Gatica, 2008; Neira & Arancibia, 2013). Additionally, considering the current impact of overfishing, data on the nutritional condition of fishery resources and their trophic relation with other species that support important fishery activities is highly relevant in the process of certification and regulation of fishing activities in the context of traceability and management in an ecological approach (FAO, 2019).

Due to the current overexploitation of the red squat lobster in the HCS, identifying how the bioenergetic condition of juvenile *P. monodon* females may vary between nursery areas of different latitudes is essential to generate sustainable-oriented management policies in fisheries with an ecological approach designed specifically for each fishing unit. Finally, recognizing the latitudinal variations in body biomass and the biochemical components of *P. monodon* juveniles and their applicability as biological tracers in the HCS is highly relevant to trace the geographic origin of the red squat lobsters that present a ”better bioenergetic condition”, all of which could greatly aid in fishery certification processes and sustainable exploitation.

## Conclusions

The differences we found in the bioenergetic condition (body mass, lipids, proteins, glucose, and energy) of juvenile females of the red squat lobster (*Pleuroncodes monodon*) from two nursery areas (NFU: 29°S *vs.* SFU: 36° S) in the HCS most likely reflect the potential physiological adaptative responses related to local habitat, which specimens have developed to face contrasting environmental factors (mainly water temperature) and coastal geomorphological characteristics of these two nursery areas. In turn, under the current overexploitation of this fishery resource in the HCS, understanding how the bioenergetic condition of juvenile *P. monodon* females may vary in nursery areas at different latitudes could help create fishery management policies designed specifically for each fishing unit. Moreover, determining the latitudinal variations of the bioenergetic condition of *P. monodon* juveniles, and using this information as biological tracers in marine ecosystems, is essential to identify the geographic origin of red squat lobsters with a “better bioenergetic condition” in the HCS, which could greatly benefit sustainable fishing certification processes.

## Supplemental Information

10.7717/peerj.13393/supp-1 Supplemental Information 1 Environmental parameters of the study area Data of sea surface temperature and chlorophyll-a off the coast of Coquimbo and Concepción, Chile

10.7717/peerj.13393/supp-2 Supplemental Information 2 Morphometric and biochemical parameters of juvenile female red squat lobster The data set indicates cephalothorax length, dry weight, lipids, protein, glucose, energy content and lipid/protein ratios of visceral and muscle of juvenile female red squat lobster from two breeding areas (off the coasts of Coquimbo and Concepción).

## Additional Information and Declarations

### Data Availability

The following information was supplied regarding data availability: The raw measurements are available in the Supplementary Files.
